# Interpretable Machine Learning for Stroke Recovery: Predicting Discharge and 3-Month Functional Outcomes

**DOI:** 10.1177/10538135261420819

**Published:** 2026-02-19

**Authors:** Inês Carvalho Martins Augusto, Nuno Antonio, Ana Marreiros, Sara Ventura Ramalhete, Hipólito Nzwalo

**Affiliations:** 1 NOVA Information Management School (NOVA IMS), Universidade Nova de Lisboa, Lisbon, Portugal; 2 Faculty of Medicine and Biomedical Sciences, Universidade of Algarve, Faro, Portugal

**Keywords:** Ischemic infarct, head injury, stroke, cerebrovascular disease, driver's rehabilitation

## Abstract

**Introduction:**

Stroke is a leading cause of disability worldwide. This study uses Machine Learning models to investigate factors influencing modified Rankin Scale scores at discharge and three months post-discharge.

**Methods:**

Data from 116 stroke patients were analyzed using four predictive models: Logistic Regression, Support Vector Machine, Random Forest, and Extreme Gradient Boosting (XGB). Shapley Additive Explanations (SHAP) were also employed to interpret factor significance.

**Results and discussion:**

The XGB model achieved an Area Under the Curve of 79% at discharge and 87% three months post-discharge. SHAP analysis revealed changing factor significance over time. The National Institutes of Health Stroke Scale was most critical at discharge, while post-discharge destination became more significant at three months. Age, time metrics, thrombolysis therapy, and management of long-term health issues also proved influential.

**Conclusions:**

Findings highlight the complex, evolving nature of stroke recovery. The shift in factor importance from clinical interventions to broader health management issues emphasizes the need for time-sensitive, multifaceted approaches to stroke care. This study contributes to understanding stroke recovery by identifying key influencing factors and demonstrating the value of SHAP for model interpretation. The insights gained have practical implications for rehabilitation practices. By identifying evolving predictors of recovery, the proposed framework may support early stratification of rehabilitation needs, assist clinicians in tailoring rehabilitation intensity and modality, and inform discharge destination decisions.

## Introduction

Stroke remains a leading cause of long-term disability worldwide (Feigin et al., 2024). Following acute hospitalization, a patient's prognosis is heavily dependent on the quality and intensity of neurorehabilitation. However, other factors also influence the functional and vital prognosis of stroke survivors, including sociodemographic characteristics (such as age, sex, and social isolation), the presence of comorbidities (Alawieh et al., 2018; Lourenço et al., 2021), in-hospital complications, and care-related processes (e.g., discharge destination and outpatient follow-up) (Nzwalo et al., 2018). Identifying these factors and understanding their relative importance are crucial for enhancing treatment, patient care, and personalized rehabilitation strategies (Yang et al., 2016).

Although conventional statistical methods are widely used in epidemiological studies and clinical trials of stroke outcomes, their reliance on predefined assumptions may limit their ability to model complex, non-linear relationships (Heo et al., 2019).

The use of Machine Learning (ML) has led to significant progress in predicting outcomes and understanding the neurobiological factors and mechanisms that distinguish poor from favorable outcomes (Bonkhoff & Grefkes, 2022). However, there is an ongoing debate regarding the trade-off between model complexity and clinical usability, as gains in predictive accuracy may come at the expense of interpretability, which is essential for clinical decision-making (Doshi-Velez & Kim, 2017; Penafiel et al., 2021).

A review of prior research reveals a heterogeneous landscape of ML applications for stroke outcome prediction (Table 1). Explainable artificial intelligence approaches, such as SHapley Additive exPlanations (SHAP), address this gap by providing transparent insights into how individual variables contribute to outcome predictions. Generalized Linear Models effectively predicted 3-month outcomes using age and NIHSS, although their performance was limited by small dataset size (Zihni et al., 2020). Other ML methods, including Artificial Neural Network (ANN), Support Vector Machine (SVM), and Deep Neural Network, have shown promise but face challenges like overfitting, limited interpretability due to their black-box nature, and reliance on single-center designs (Asadi et al., 2014; Heo et al., 2019). In addition, Deep Neural Networks showed efficacy in predicting long-term outcomes but faced limitations due to a single-center study design (Heo et al., 2019). Logistic Regression and Random Forest identified key predictive and prognostic factors, though missing data and limited parameter space were constraints (Wang et al., 2023; Zhang, 2013). Evidence from most studies indicates that integrating human expertise with AI, emphasizing interpretability, and carefully balancing accuracy with usability represents the most effective approach (Bonkhoff & Grefkes, 2022; Penafiel et al., 2021).

**Table 1.** Features of Prior Research.

Study	Outcome Timepoint	Models Used	Interpretability Issues	Reported Key Predictors	Main Limitation Reported
Zihni et al. (2020)	3-month	GLM	Coefficients	Age, sex, NIHSS, cardiac history, diabetes mellitus, hypercholesterolemia, thrombolysis.	Dataset constraints
Asadi et al. (2014)	3-months	ANN, SVM	“Black-box”	NIHSS, stroke subtype, age, type II diabetes, general anesthesia, previous stroke, thrombolysis in cerebral infraction scale.	Overfitting/interpretability
Heo et al. (2019)	3-months	DNN	Limited	Demographics, NIHSS, time from onset to admission, stroke subtype, clinical history.	Single-center study design
Zhang (2013)	48-h	LR	Coefficients	Demographics, physiological parameter	Limited parameters
Wang et al. (2023)	1-year	Random Forest	SHAP	Neuron specific enolase, homocysteine, S-100β, dysphagia, C-reactive protein, anticoagulation.	Missing NIHSS

This study addresses that gap by analyzing changes in prognostic factors from discharge to three months post-discharge, with the goal of enhancing the transparency and clinical utility of ML models for stroke prognosis. Unlike prior publications that focused on single timepoints or prioritized black-box accuracy, our approach delivers an interpretable, time-sensitive analysis that reveals a clinically actionable shift in importance, from acute severity to post-discharge contextual factors.

The study had two main objectives: identifying key factors influencing stroke recovery outcomes after stroke and developing an ML model that balances accuracy with interpretability. Additionally, the study was designed to address the following research questions: (1) how do predictive models perform in classifying patient modified rankin scale (mRS) scores, particularly in differentiating between favorable and unfavorable prognoses?; (2) what are the key factors influencing mRS scores?; and (3) what are the temporal trends of these factors from discharge to three months post-stroke? Unlike prior studies that focus on static time points, this study investigates the temporal evolution of prognostic factors from discharge to three months post-stroke using interpretable ML techniques.

## Methods

Data was collected from 125 hospitalized stroke patients during the Spring of both 2019 and 2020 (March, April, May). Following exploratory data analysis, a cleaning process was conducted that removed duplicate episodes and those lacking information. Missing values were addressed through imputation based on the distributions of the respective variables. Variables with more than 50% missing data were excluded from the analysis. As a result, data from 116 patients were analyzed (Table 2), focusing on variables related to health behaviors, medical conditions, treatments, and stroke severity (Alawieh et al., 2018; Chen et al., 2025). All analyses were conducted in Python using packages, including the scikit-learn (Pedregosa et al., 2011). Moreover, to ensure reproducibility and transparency of the study, the following details were reported: study population and setting, patient demographic characteristics, model architecture, and model evaluation (Hernandez-Boussard et al., 2020).

**Table 2.** Features Summary Statistics After Data Cleaning.

Feature Name	Count	Mean	Std	Min	0.25	0.5	0.75	Max	Description
Age	116	70.78	13.61	27	62	74	80	97	Age in years
Sex	116	0.56	0.50	0	0	1	1	1	Sex (Male/Female)
HTN	116	0.81	0.39	0	1	1	1	1	If the patient has high blood pressure
Diabetes	116	0.28	0.45	0	0	0	1	1	If the patient has diabetes
Dyslipidaemia	116	0.62	0.49	0	0	1	1	1	If the patient has dysplidaemia
Smoking	116	0.22	0.42	0	0	0	0	1	If the patient is smoker
Obesity	116	0.16	0.36	0	0	0	0	1	If the patient is obese
Auricular Fibrillation	116	0.16	0.37	0	0	0	0	1	If the patient has auricular fibrillation
Anti-aggregation	116	0.17	0.38	0	0	0	0	1	If the patient takes anti-aggregation medicines
Anti-coagulation	116	0.12	0.33	0	0	0	0	1	If the patient takes anti-coagulation medicines
Oxfordshire	116	1.07	1.08	0	0	1	2	4	Oxfordshire classification
NIHSS	116	8.65	7.08	0	3	6	12.25	25	NIHSS scale
Glasgow	116	13.76	1.82	9	13	15	15	15	Glasgow scale
Time symptoms-door	116	486.84	962.20	0	61.5	119	513.75	6480	Minutes since symptoms to hospital
Time door-TAC	116	87.73	87.68	0	36	55	104.5	560	Minutes since hospital admission to TAC analysis
Diagnosis	116	0.54	1.14	0	0	0	1	5	Diagnosis of stroke patient
Patient transferred	116	0.10	0.31	0	0	0	0	1	If the patient was transferred to another hospital
Thrombolysis	116	0.24	0.43	0	0	0	0	1	If the patient underwent thrombolysis
Thrombectomy	116	0.10	0.31	0	0	0	0	1	If the patient underwent thrombectomy
TOAST	116	1.45	1.42	0	0	1	2.25	4	TOAST classification
Destination	116	0.36	0.69	0	0	0	1	3	Discharge destination
Month	116	3.71	0.53	3	3	4	4	5	Month of the event
Year	116	2019.34	0.47	2019	2019	2019	2020	2020	Year of the event

To run the model on the day following the admission date, the variable “Destination” was removed from all models predicting mRS at discharge. This decision was made because the necessary information was not obtainable on the day after the admission.

Afterwards, a correlation analysis was performed to identify factors associated with mRS scores. The *p*-value was used to assess the statistical significance of the correlations, with a smaller *p*-value suggesting that the observed relationships were less likely to be due to chance. For the analysis, a *p*-value threshold of 0.05 was considered.

The Kruskal-Wallis test was performed to identify if there was a statistically significant difference in the two mRS scores between patients with or without obesity. In addition, Dunn's test was conducted to determine if stroke subtype and obesity were statistically associated with both scores. These analyses were motivated by previous studies that identified an association between obesity and mRS score (Oesch et al., 2017).

Moreover, we employed supervised learning algorithms, including LR, SVM, Random Forest (RF), and Extreme Gradient Boosting (XGB), focusing on the target mRS, ranging from 0 to 6, at discharge (mRS D) and three months later (mRS 3M) (Higashida & Furlan, 2003; Pedregosa et al., 2011). To align with the study objectives, both target variables were binarized into two groups: mRS ≥ 3, indicating a poor prognosis (labeled as 0), and mRS < 3, indicating a good prognosis (labeled as 1). This cut-off was defined as an mRS 0-2, which is usually considered a good functional outcome (Weisscher et al., 2008). Additionally, to ensure the model could be applied on the day following admission, the variable destination was excluded from all models targeting mRS at discharge, as it would not be available at that time.

Additionally, the presence of numerous features and a limited number of data points in this dataset posed significant challenges for the model's performance. To address this issue, Recursive Feature Elimination (RFE) was employed to select the most relevant features and enhance the model's effectiveness (Pedregosa et al., 2011).

Thus, RFE was used to select features for LR and SVM models for predicting mRS at discharge. The LR model selected two features: Age and NIHSS, while the SVM model selected five features: HTN, Dyslipidemia, National Institutes of Health Stroke Scale (NIHSS), Glasgow, and Trial of Org 10172 in Acute Stroke Treatment (TOAST). For predicting mRS at three months post-discharge, the LR model selected NIHSS and Destination, whereas the SVM model selected four features: Age, NIHSS, Time door-to-CT, and Destination.

Preprocessing steps were tailored to the model architecture. Feature scaling using a MinMax scaler was applied to linear and distance-based models (LR and SVM). In contrast, tree-based models, which are invariant to monotonic feature transformations, were trained on unscaled data. Although LR and SVM do not require bounded input data specifically, normalizing the data can significantly improve the effectiveness of these models. This approach leads to a more stable and faster training process and can result in better overall model performance.

To overcome the shortage of labelled data for an independent validation set, we performed 10-fold cross-validation (CV). It was observed that the distribution of the target variable's values was imbalanced. Since this issue affects LR and SVM models more, the Synthetic Minority Oversampling Technique (SMOTE) was applied to the training dataset to address it (Chawla et al., 2002). Additionally, we used the GridSearch function to optimize the parameters for our four ML models, and the selected parameters (Table 3) (LaValle et al., 2004).

**Table 3.** Final Parameters for Each Model.

Model	GridSearch Tested Values	Parameters (mRs At Discharge)	Parameters (mRS At Three Months)
LR	C = {0.01, 0.05, 0.1, 0.5, 1}; Solver = {lbfgs, liblinear} Max_iter = 100 N_features = {2:20}	C = 1; Solver = liblinear; Max_iter = 100; N_features = 2	C = 0.1; Solver = liblinear; Max_iter = 100; N_features = 2
SVM	C = {0.1, 0.2,0.3,0.4, 0.5, 1} Class_weight = balanced; Kernel = linear	C = 0.5; Class_weight = balanced; Kernel = linear	C = 1; Class_weight = balanced; Kernel = linear
RF	n_estimators = {3,4,5,6,7,8,9,10}; criterion = {gini, entropy} max_depth = {2,3,4} min_samples_split = {2,3} min_samples_leaf = {1,2,3} class_weight = balanced	n_estimators = 10; criterion = gini; max_depth = 2; min_samples_split = 2; min_samples_leaf = 1; max_features = 8; class_weight = balanced	n_estimators = 10; criterion = entropy; max_depth = 3; min_samples_split = 2; min_samples_leaf = 2; max_features = 4; class_weight = balanced
XGB	max_depth = {2, 3, 4} learning_rate = {0.01, 0.05, 0.1} n_estimators = {7, 8, 9, 10} subsample = {0.7, 0.8, 0.9} colsample_bytree = {0.7, 0.8, 0.9}	max_depth = 2; learning_rate = 0.05; n_estimators = 10; subsample = 0.8; colsample_bytree = 0.9	max_depth = 3; learning_rate = 0.01; n_estimators = 10; subsample = 0.8; colsample_bytree = 0.9

Because the LR model is susceptible to outliers, we used an isolation forest to detect and handle them. However, this led to more overfitting in the training and test sets. To address this, different solvers and regularization parameters were tested (Table 3). For SVM, we tried several kernels, penalties, and other methods to address class imbalance and found that SMOTE yielded the best results. In RF, the parameter class_weight = balanced was added. Similarly, in XGB, we used the scale_pos_weight parameter. To optimize our XGB model, we began by using GridSearchCV to tune the training and test splits. However, we eventually tried different parameters and manually fine-tuned them multiple times to achieve the final metrics (Table 3). Additionally, we implemented early_stopping_rounds = 10 to prevent overfitting.

Moreover, the performance of the models was assessed on both the training and test sets using the average of the following metrics: accuracy, AUC, and recall.

Finally, although we selected the models based on previous studies to ensure the validity of our results, unlike those studies, we used Shapley Additive Explanations (SHAP) to highlight feature impacts and improve interpretability and assessment of feature contribution (Zihni et al., 2020).

## Results and Discussion

### Factors Associated with mRS Score

A correlation matrix was developed to identify the factors associated with mRS, but no variables were correlated with more than 0.5 points (Table 4). These relationships can be divided into two groups: a feature can positively or negatively correlate with the target variables. In other words, a positive relationship occurs when the variables increase, and the target variables increase as well. On the other hand, a negative correlation occurs when, as one variable increases, the other decreases.

**Table 4.** Correlation Analysis Between Socio-Demographic, Clinical and Comorbidities and mRS at Discharge and 3-Months of Discharge.

Feature Name	mRS At Discharge		mRS At Three Months of Discharge	
	Correlation	*p*-value	Correlation	*p*-value
Age	0.16	0.08	0.2	0.03
Sex	−0.13	0.17	−0.03	0.73
HTN	0.14	0.15	0.14	0.15
Diabetes	0	0.98	−0.02	0.8
Dyslipidaemia	−0.08	0.41	−0.07	0.48
Smoking	−0.15	0.12	−0.08	0.39
Obesity	−0.17	0.06	−0.14	0.12
Auricular Fibrillation	−0.04	0.65	0.11	0.24
Anti-aggregation	−0.06	0.53	−0.01	0.92
Anti-coagulation	0	0.97	0.02	0.81
Oxfordshire	0.05	0.58	0.05	0.57
NIHSS	0.46	0	0.3	0
Glasgow	−0.24	0.01	−0.2	0.03
Time symptoms-door	−0.03	0.77	0.05	0.63
Time door-TAC	−0.01	0.92	0.05	0.57
Diagnosis	−0.06	0.52	−0.11	0.24
Patient transferred	0.1	0.3	0.12	0.19
Thrombolysis	−0.01	0.95	−0.1	0.27
Thrombectomy	0.1	0.3	0.06	0.54
TOAST	0.13	0.17	0.05	0.6
Destination	N/A	N/A	0.49	0
Month	0.01	0.91	−0.08	0.38
Year	−0.04	0.65	0.16	0.08

In the context of positive relationships, patients with higher mRS scores are more likely to have outcomes that align with higher fate values, such as the need for care in a specialized unit or nursing home, or, in the worst cases, result in death. Another example is the fact that a higher NIHSS score indicates more severe neurological impairment, which is often associated with more significant disability or dependence, as reflected by a higher mRS score. Similarly, older patients often have worse outcomes after a stroke or other neurological events, with the conclusion that the older the patient, the higher their mRS score.

On the contrary, higher GCS values, which indicate milder impairment, are associated with lower mRS values. This association implies that patients who are more alert and reactive, as measured by the GCS, tend to have better functional outcomes, as reflected by lower mRS scores. Regarding the diagnosis variable, the negative correlation indicates that certain stroke types or related conditions are associated with lower mRS scores. For example, hemorrhagic stroke may be associated with higher mRS scores.

**Figure 1.**
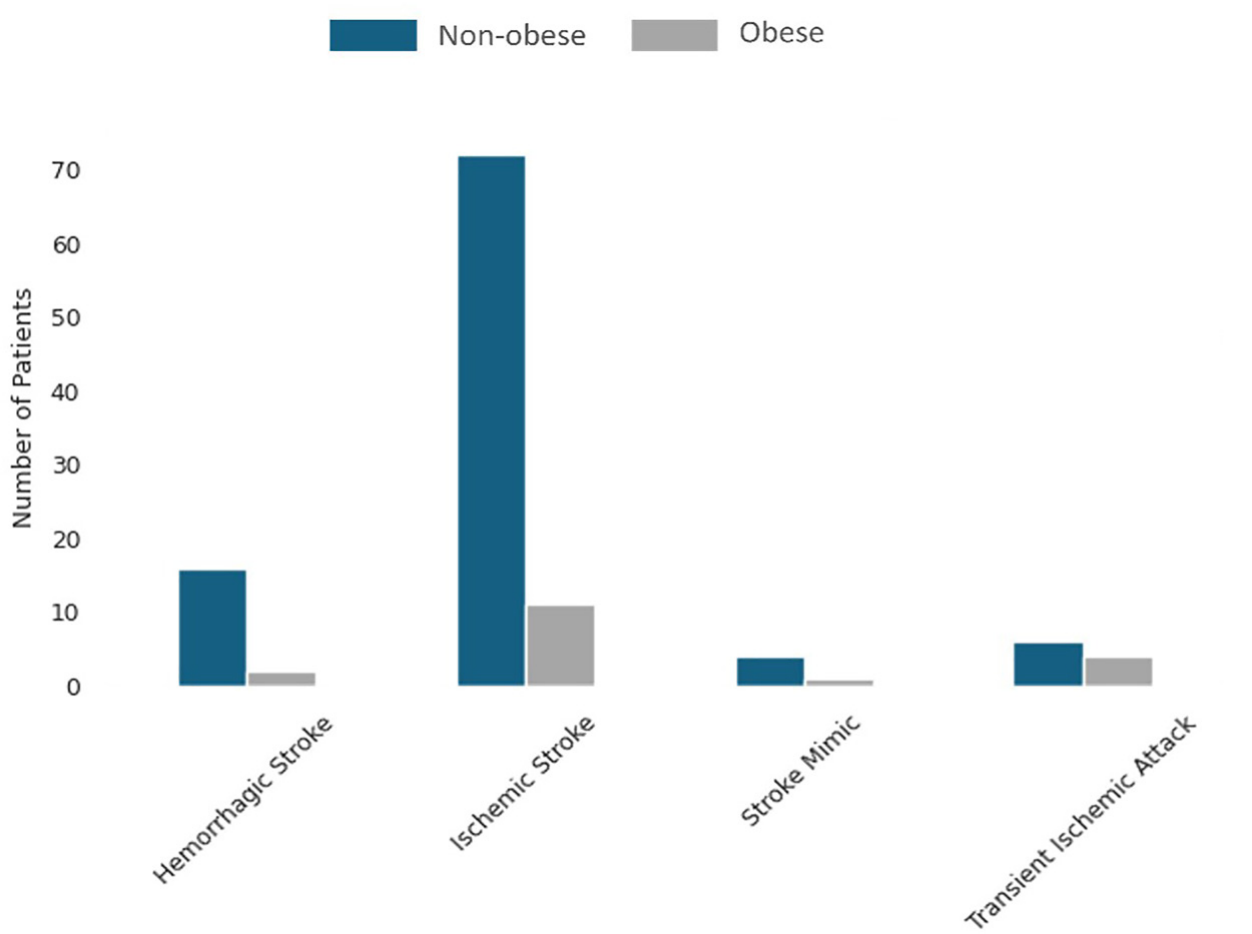
Distribution of stroke types by obesity status.

Finally, the obesity variable suggests that non-obese patients may have worse functional outcomes, which seems counterintuitive given that obesity is often associated with worse health conditions. Oesch et al. (2017) identified a strong and consistent “obesity paradox” across multiple studies, where obese patients exhibit better survival rates and recovery outcomes after a stroke. Moreover, Kruskal-Wallis and Dunn's tests revealed that obese patients suffered predominantly ischemic strokes, which generally have more favorable recovery outcomes compared to hemorrhagic strokes (Figure 1).

### Machine Learning Analysis

**Figure 2.**
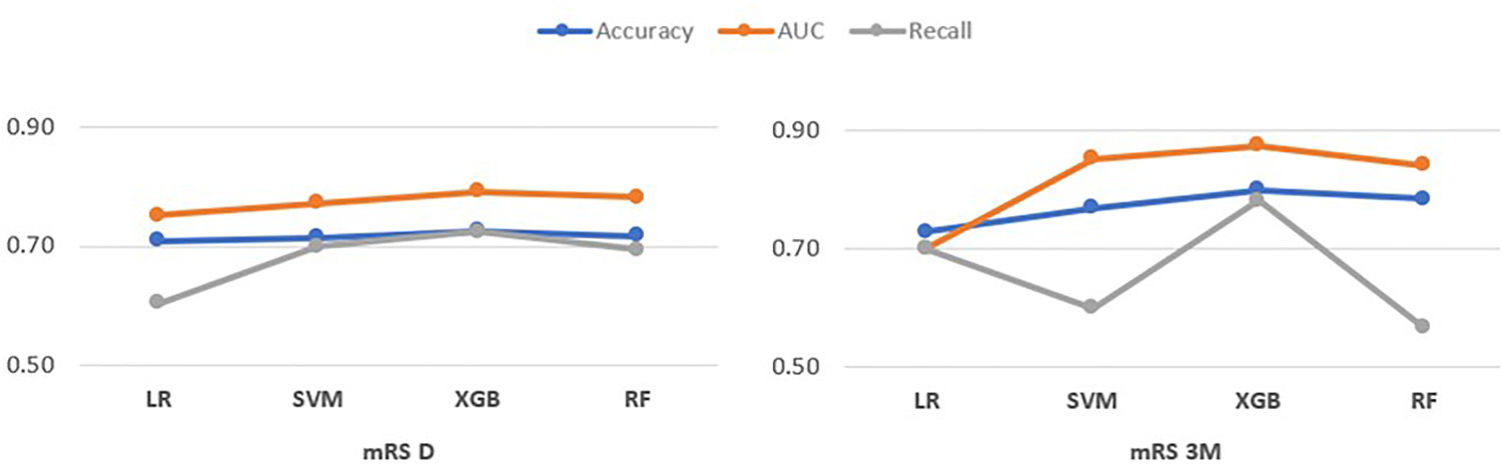
Evaluation metrics in model test sets.

The four ML models were applied to the dataset. The XGB model performed best, with AUCs of 79% for mRS D and 87% for mRS 3 M. SVM had an AUC of 77% for mRS D and 85% for mRS 3 M. RF had 78% and 84% AUCs for mRS D and mRS 3 M, respectively. Finally, LR obtained 75% and 70% AUCs for mRS D and mRS 3 M (Figure 2).

LR and SVM showed similar performance across both target variables, indicating their stability across different time frames for this dataset. The XGB model showed a drop in recall for mRS 3 M, suggesting that it may need to be more effective at identifying true positives at three months, as at discharge. Similarly, the RF model showed consistent performance for mRS D but had a drop in recall for mRS 3 M, similar to XGB. The analysis also revealed that the mRS D predictions were more consistently accurate across all models than the mRS 3 M predictions. This result could imply that predicting outcomes at discharge was a simpler task because there was less variability or noise in the data at that time point. Many additional post-discharge factors likely affect mRS 3 M predictions, making the task more challenging.

As previously mentioned, the obesity variable showed a negative correlation with both target variables. It may seem unexpected, but these findings could be affected by the greater number of non-obese patients in the dataset, potentially biasing the statistical analysis. Furthermore, according to the results of the Kruskal-Wallis test, there was no statistically significant difference between the obese and non-obese groups for both mRS D (*p*-value = 0.06, H-statistic = 3.46) and mRS 3 M (*p*-value = 0.12, H-statistic = 2.40). However, the near-threshold significance for mRS D might merit further study.

Likewise, in Dunn's test, there was no evidence suggesting that obesity has a statistically significant impact on mRS D and mRS 3 M for any of the analyzed stroke types (Table 5). This finding was consistent with the Kruskal-Wallis test results, which showed an overall difference that was borderline significant but inconclusive.

**Table 5.** Results of Dunn's Test.

	Ischemic Stroke	Haemorrhagic Stroke	Stroke Mimic	Transient Ischemic Attack
H-statistic	1.36	0.27	0.25	0.67
*p*-value	0.24	0.61	0.62	0.41

**Figure 3.**
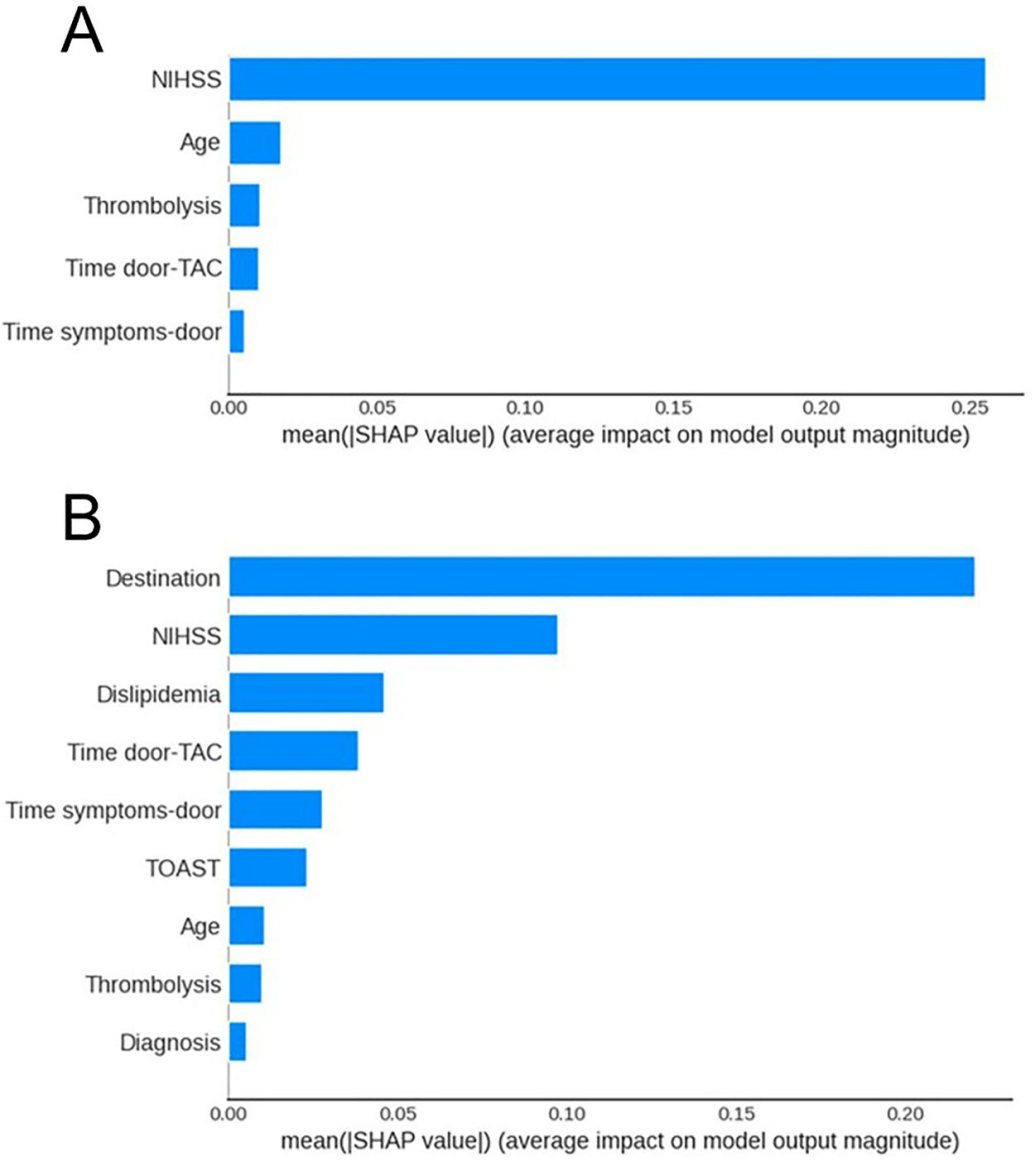
Importance ranking of features on (A) mRS D and (B) mRS 3M.

**Figure 4.**
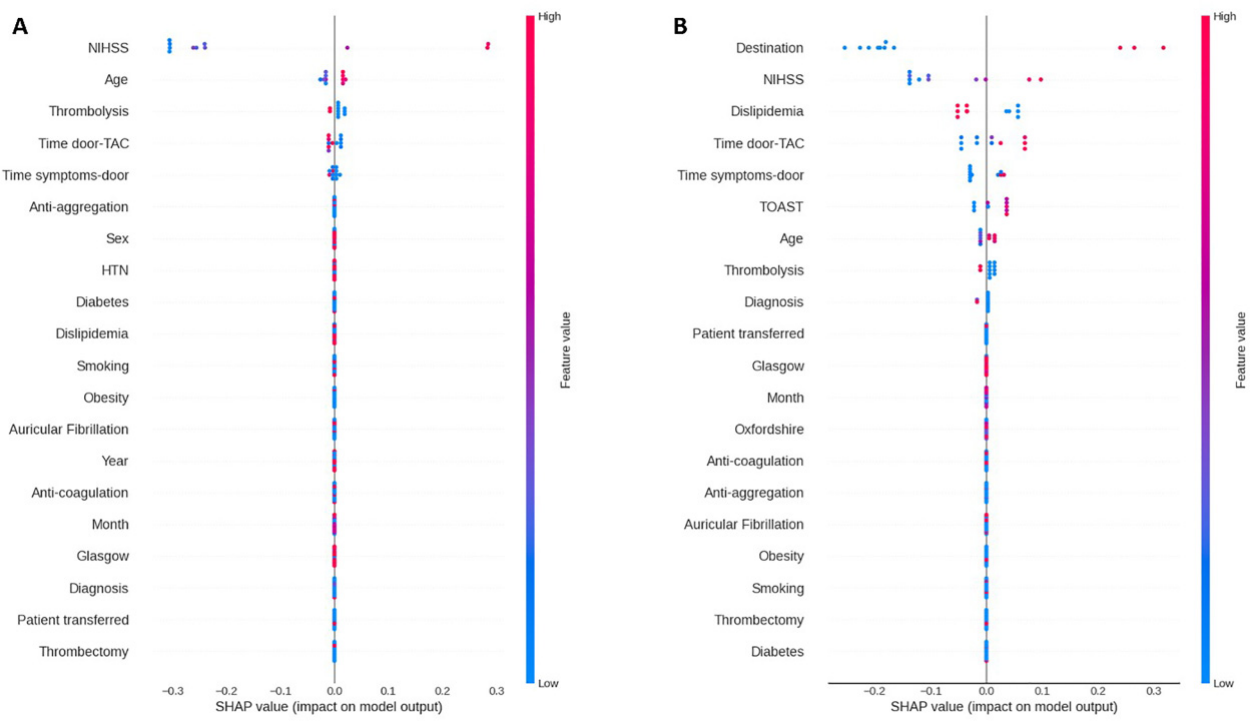
Impact of each feature (SHAP value). (A) on mRS D. (B) on mRS 3M.

Finally, we applied SHAP analysis to interpret the XGB model's results, which achieved the highest AUC for classifying patient prognosis. This analysis was used not only to assess feature importance but also to examine its temporal progression, offering new insights into how prognostic influences shift throughout the stroke recovery timeline. The SHAP results for the XGB model revealed the most significant factors affecting mRS scores and their temporal trends (Figures 3 and 4). The pattern changed slightly in the final period, with additional important features emerging.

Our study revealed that NIHSS and age consistently predicted immediate and short-term disability outcomes, consistent with previous studies (Asadi et al., 2014; Heo et al., 2019; Zihni et al., 2020). Additionally, time metrics emerged as influential factors, highlighting the importance of prompt medical intervention. This finding aligns with previous studies, which emphasize the importance of timely admission (Heo et al., 2019). For instance, patients with higher NIHSS scores or advanced age may require more intensive, individualized rehabilitation strategies to optimize recovery. At the same time, early intervention and rapid hospital admission can enhance the effectiveness of rehabilitation programs by reducing the extent of initial neurological damage and integrating these predictive factors.

The mRS D was influenced by patients’ post-discharge destinations, emphasizing the impact of post-acute care environments. This factor, rarely discussed in literature, emphasizes the need for effective post-discharge planning. Our study also highlighted new factors, including dyslipidemia, the importance of thrombolysis, diagnosis, and the TOAST classification, underscoring the multifaceted nature of stroke recovery.

Trends from discharge to three months after discharge reflect a shift from acute clinical interventions to broader health management issues. Initial stroke severity and age continue to have a significant influence, while the emphasis increases on managing long-term health problems and ensuring continuity and appropriateness of care after discharge. Effective, timely, and sustained rehabilitation interventions are critical for translating early neurological stability into functional recovery, particularly as patients transition from hospital-based care to community or outpatient settings. Ensuring the appropriateness and coordination of post-discharge rehabilitation services may therefore mitigate disability progression and support improved functional independence in the subacute recovery period. Furthermore, our results confirm the conclusions of previous studies. For instance, emphasizing the significant impact of comorbid conditions on stroke recovery, highlighting the complexity of health problems (Demchuk & Buchan, 2000). Another study found greater diversity in less critical features, consistent with our discoveries (Zihni et al., 2020). Additionally, a study highlighted the crucial link between stroke characteristics and the effectiveness of clinical interventions, underscoring their fundamental role in shaping recovery outcomes (Banks & Marotta, 2007). Notably, we identified new factors influencing stroke recovery, highlighting the complexity and variability of clinical data.

## Conclusions

In this study, we proposed a predictive model that achieved good discriminative performance for functional outcome prediction, with an AUC of 79% at discharge and 87% at three-month post-discharge. Beyond predictive accuracy, integrating SHAP analysis provided substantial added value by revealing time-dependent changes in the relative importance of prognostic factors. This approach extends beyond earlier ML-based studies that focused on a single timepoint or relied on static feature importance. Indeed, with strategy, we underscored the dynamic nature of recovery, as the relative importance of prognostic factors evolves over time, with clinical and socio-environmental factors exerting influence at different stages.

These insights have direct implications for stroke rehabilitation practices. By identifying evolving predictors of recovery, the proposed framework may support early stratification of rehabilitation needs, assist clinicians in tailoring rehabilitation intensity and modality, and inform discharge destination decisions. In particular, the strong influence of post-discharge context highlights the importance of coordinated post-acute care pathways and timely access to rehabilitation services in promoting functional recovery.

Moreover, given that all relevant data can be easily obtained from clinical electronic records, automatic alert systems (pop up in electronic charts) could be implemented to notify providers of at-risk patients, thereby facilitating the allocation of more intensive neurorehabilitation.

This study also significantly contributes to the literature on stroke outcome prediction by identifying key factors for recovery and by showcasing SHAP values to interpret ML models. By examining temporal shifts in prognostic relevance from discharge to three months, our approach extends beyond earlier studies that focus on a single time point or rely on static feature importance. In particular, identifying under-reported predictors, such as discharge destination, broadens the scope of stroke prognosis beyond purely acute clinical measures. It underscores the importance of post-acute care pathways.

The findings highlight practical implications for healthcare, highlighting the need for rapid intervention and comprehensive data analysis. These insights can inform more accurate predictive models, improving patient management and treatment strategies. Also, the model has the potential to inform personalized rehabilitation strategies by identifying key determinants of recovery, thereby supporting clinicians in optimizing therapy intensity, modality selection, and post-discharge care planning. Nevertheless, the study's retrospective design may introduce selection bias, and the small sample size (*n* = 116) limits generalizability. Data from a single medical center also affects external validity. Future research should incorporate additional data sources, such as imaging, to strengthen model interpretability and robustness. Additionally, future work should focus on federated and domain-adaptive learning strategies to enable privacy-preserving, multi-institutional validation and improve robustness across heterogeneous clinical settings. The small sample size (*n* = 116) also increases the risk of overfitting and instability in model selection despite the validation strategy employed. Hyperparameter optimization was limited to a constrained search for transparency and to avoid over-tuning on a small cohort. Therefore, additional performance gains may be possible through more systematic optimization and external validation on larger datasets. It is also important to note that the complex interplay of clinical factors, which underlies ML (Asadi et al., 2014; Castro et al., 2025; Wang et al., 2023), could not be fully explored, as many variables were unavailable.

ML algorithms excel in identifying correlated features but face challenges in predicting outcomes due to the complex interplay of clinical factors (Asadi et al., 2014; Wang et al., 2023). It is important to note that while these models predict outcomes, they do not establish causation (Obermeyer & Emanuel, 2016). The lack of standardized baseline models (e.g., unregularized logistic regression) limits the ability to quantify the incremental benefit of the advanced ML pipeline. Future work should include such baselines and external validation.

Although RFE improved model parsimony, it does not explicitly mitigate multicollinearity, and correlated predictors may remain in the final set. Moreover, the proposed model does not explicitly account for temporal dependencies between acute and post-discharge assessments, nor does it systematically test interactions between predictors, which may influence stroke recovery trajectories. These aspects should be explored in future work using time-aware modeling and interaction-based approaches.
